# Effects of stress after road transportation and oral administration of chromium and meloxicam on plasma cortisol concentrations and behavior in dairy calves

**DOI:** 10.5713/ab.21.0321

**Published:** 2021-10-29

**Authors:** Da Jin Sol Jung, Jaesung Lee, Do Hyun Kim, Seok-Hyeon Beak, Soo Jong Hong, In Hyuk Jeong, Seon Pil Yoo, Jin Oh Lee, In Gu Cho, Dilla Mareistia Fassah, Hyun Jin Kim, Myunggi Baik

**Affiliations:** 1 Department of Agricultural Biotechnology and Research Institute of Agriculture and Life Sciences, Seoul National University, Seoul 08826, Korea; 2 Institutes of Green Bio Science Technology, Seoul National University, Pyeongchang 25354, Korea

**Keywords:** Chromium, Dairy Heifer, Meloxicam, Transportation Stress

## Abstract

**Objective:**

This study was performed to determine the effects of stress after road transportation and oral administration of chromium and meloxicam on growth performance, plasma cortisol, serum metabolites, and behavior in dairy calves.

**Methods:**

A total of 50 Holstein heifers (average body weight [BW]: 172±4.19 kg; average age: 5.53±0.12 months) were randomly assigned to five groups including NL (not transported + D-lactose; 1 mg/kg BW), TL (transported + D-lactose; 1 mg/kg BW), TC (transported + chromium; 0.5 mg/kg dry matter [DM] feed), TM (transported + meloxicam; 1 mg/kg BW), and TMC (transported + combination of meloxicam and chromium; 1 mg/kg BW and 0.5 mg/kg DM, respectively). Doses of D-lactose monohydrate, meloxicam, and chromium were prepared for oral administration by suspension in 15 mL of water in a 20-mL dosing syringe. Blood was collected before transportation, immediately after 120 km of transportation (IAT), and at 6, 24, and 48 h after transportation.

**Results:**

Neither transportation nor administration of meloxicam and/or chromium affected (p = 0.99) average daily gain and feed intake. Plasma cortisol concentrations in the NL group (average 0.13 and 0.18 nmol/L, respectively) were lower (p<0.001) compared to the TL group (average 0.39 and 0.61 nmol/L, respectively) at IAT and 48 h after transportation. At 48 h after transportation, cortisol concentrations were lower (p<0.05) in the TC group (average 0.22 nmol/L) than in the TL group (average 0.61 nmol/L), and TC calves had similar cortisol concentrations to NL calves. Lying duration (min/d) was shorter (p<0.05) in the TL group than in the NL group at 2 d after transportation. Lying duration was longer (p<0.05) for the TC and TMC groups than for the TL group at 2 d after transportation.

**Conclusion:**

Transportation increased cortisol concentrations and affected lying behavior, while chromium administration reduced cortisol concentrations and changed lying behavior. Thus, chromium administration before transportation may be a viable strategy to alleviate stress elicited by road transportation.

## INTRODUCTION

Dairy farms in South Korea usually work on milking lactating cows and rearing heifers simultaneously. However, this system is inefficient for dairy farmers due to the lack of space for rearing lactating cows and labor constraints associated with raising heifers. Commercial heifer-raising farms have been established to solve these problems. From 3 months of age, dairy calves are sent by dairy farmers to commercial heifer-raising farms, grown, inseminated, and then returned to the original dairy farm at around 8 months of pregnancy. Transportation distance of cattle in South Korea is relatively short compared to other foreign countries because cattle are usually moved within local province. In our previous study, relatively short-distance (both 100 km and 200 km) of road transportation caused a transient increase in circulating cortisol and changes in the metabolic and immunological parameters in Holstein-heifers [1]. Thus, we performed 120 km of short-distance road transportation in this study.

Road transportation causes inevitable stress when operating commercial heifer-raising farms. Transportation can induce inflammatory and acute-phase responses in cattle [2], disturb animal behavior, and retard animal growth [3,4]. Circulating cortisol is widely used as the main indicator of stress, since cortisol secretion is activated by various stresses via the hypothalamic–pituitary–adrenal (HPA) axis [5]. The body energy and protein imbalance during the transportation period are influenced by the HPA axis, resulting in the secretion of circulating cortisol [6]. Consequently, transportation may lead to physiological and psychological changes such as body shrink or dehydration [7], decreased feed intake [8], tissue damage [9], and changes in animal behaviors in cattle. Therefore, appropriate management through stress reduction strategies is necessary for improving animal welfare and health.

Among many stress reduction strategies, chromium (Cr) was effective at regulating glucose metabolism by increasing insulin sensitivity under stress conditions [10]. A high forage diet supplemented with the Cr group had higher circulating insulin levels compared with the control group in Korean cattle steers [11]. Cr can also reduce heat stress in dairy cows [12]. Limited information is available on the effects of Cr on transportation stress. Nonsteroidal anti-inflammatory drug (NSAID) can reduce stress and an inflammatory response and improve cattle performance by inhibiting both cyclo-oxygenase-1 (COX-1) and COX-2 enzymes. Meloxicam is one of NSAIDs that selectively inhibits the synthesis of the COX-2, which is more closely related to inflammatory response. Several studies have confirmed that NSAID administration before transportation, castration, or dehorning reduces circulating cortisol in cattle [13–15].

We hypothesized that meloxicam and Cr administration before transportation would affect circulating cortisol and glucose concentrations and behavior of the transported calves. This study investigated the effects of stress after road transportation and administration of Cr and meloxicam on growth performance, cortisol and glucose concentrations, and behavior in transported dairy calves.

## MATERIALS AND METHODS

### Animals

All experimental procedures involving animals were approved by the Seoul National University Institutional Animal Care and Use Committee (SNUIACUC: SNU-180717-2) and conducted in accordance with the Animal Experimental Guidelines of the SNUIACUC. This experiment was conducted at a heifer-specific farm located at 825–7 in Mussu-ri, Dangjin, South Chungcheong Province, South Korea.

A total of 50 Holstein heifers (average body weight [BW] 172±4.19 kg; average age 5.53±0.12 months) were randomly assigned to five groups: NL (no transportation + D-lactose administration; 1 mg/kg BW), TL (transportation + D-lactose administration; 1 mg/kg BW), TC (transportation + Cr administration; 0.5 mg/kg dry matter [DM] feed), TM (transportation + meloxicam administration; 1 mg/kg BW), and TMC (transportation + Cr and meloxicam administration; 0.5 mg/kg DM feed and 1 mg/kg BW, respectively).

The calves were fed 8 kg of total mixed ration, including 26.55% concentrate. The formula and chemical composition of the diet are shown in Table 1 and 2. Fresh water was available *ad libitum*.

Initial BW and final BW of heifers were measured at −1 d and 14 d to calculate average daily gain, feed intake, and gain:feed. During the experimental period, all heifers were fed the total mixed ration. The fixed amount of total mixed ration was offered at 0800 and 1400 h daily to each pen. Group intakes were recorded daily throughout the study by weighing the amount of feed offered and the amount of feed refused. These data were used to calculate daily intake per pen.

The heifers were adapted in a stanchion barn. All heifers were calmed in a stanchion before transportation. All heifers were fasted for 9-h before the first blood sampling and kept fasting until the end of transportation. No feed or water was provided to the NL group when other groups were transported. After transportation, feed and water were provided to all heifers. The animals were fasted again 9 h before blood sampling at 1 and 2 d after transportation.

### Cr and meloxicam administration

Oral doses of D-lactose monohydrate, Cr, and meloxicam were prepared by suspension in 15 mL water in a 20-mL dosing syringe, then administered 2 h before transportation. We chose this timing for oral administration because peak blood meloxicam concentration is 3 to 8 h after oral administration [16,17]. Heifers in the placebo group were administered D-lactose monohydrate (1 mg/kg BW; Avantor Performance Materials, Center Valley, PA, USA), which is a pharmaceutically acceptable inactive excipient used in the manufacture of meloxicam capsules. Most of Cr supplementation studies evaluated with 0.3 to 0.5 mg/kg DM per day [18]. Thus, in this study, the Cr was orally administered as chromium picolinate at 0.5 mg/kg DM (Samjo Life Science, Seoul, Korea). It has been suggested that a single dosage of oral meloxicam at 1 mg/kg BW can reduce the stress in ruminant calves [16], and oral meloxicam administration at 1 mg/kg BW was partially effective for reducing the impact of long-distance transportation on physiological biomarkers of stress in beef calves [15]. Based on these literatures, the meloxicam (MOBIC CAP, 15 mg; Boehringer Ingelheim, Ingelheim am Rhein, Germany) was orally administered at 1 mg/kg BW in this study.

### Transportation

The transportation began at 12:00 pm in May 2019 when the daily average temperature and humidity were 24.1°C and 43.0%, respectively. The trucks, used exclusively for cattle transportation, were prepared following Korean animal welfare standards. The trucks could transport a maximum of 10 cattle with 1.84 m^2^/animal and had a proper cover for wind and sun protection as well as a ventilation system on both sides of the truck. Four trucks were used for transportation, and each truck transported 10 heifers while the NL heifers remained in the feedlot. Loading and unloading process of heifers were carried out safely and well guided by well-trained handlers. Tailgate of the truck bed, which was specially designed for cattle loading and unloading, was lowered so that the heifers could easily ride on the truck. Feedlot fences and awning screens were used to create a path for heifers to truck, and cattle were loaded or unload through the path. The transportation was 120 km round-trip from the starting point at a driving speed of 65 km per hour. All trucks departed simultaneously and moved in a line until the end of transportation. Transportation trucks drove on a highway except for 12.4 km of local roads between the experimental farm and highway. Animals were returned to their stanchions immediately after transportation (IAT).

### Blood collection and analysis

Blood samples were collected 2.5 h before transportation, IAT, and at 6, 24, and 48 h after transportation. The blood samples were collected by jugular venipuncture using both non-heparinized vacutainers (20 mL; BD Biosciences, San Jose, CA, USA) and ethylenediaminetetraacetic acid-treated vacutainers (20 mL). Blood samples were immediately placed in an icebox. The serum and plasma were separated by centrifugation at 1,500×g for 15-min at 4°C. The plasma and serum were subsequently stored at −80°C until analysis.

Serum glucose was analyzed with a fully automated Cobas 8000 C702 analyzer (Roche Diagnostics, Mannheim, Germany) using colorimetric methods with specific kits. A Roche GLUC2 kit was used for the analysis of serum glucose (Glucose HK Gen.3; Roche Diagnostics, Germany). Plasma cortisol was analyzed using a salivary cortisol enzyme immunoassay kit (Salimetrics, State College, PA, USA). The coefficient variances of the intra-assay and inter-assay of the cortisol kit for bovine plasma samples were 4.2% and 4.8%, respectively. The analytical method for cortisol assay was validated in previous report from our laboratory [1].

### Lying behavior observation

Lying behavior (duration of lying; min/d) was video-recorded from 9 am to 8 pm on 2 days (1 and 2 d after transportation) using video cameras (C3S; Ezviz, Hangzhou, China) positioned approximately 8 m above each experimental pen. The recordings were stored on a 128 GB micro SD memory card (Sony Corporation, Tokyo, Japan). Individual animals were identified with unique colors by body taping.

### Statistical analyses

All data except growth performance were analyzed as a completely randomized design using repeated measures of the MIXED procedure in SAS (SAS Institute, Cary, NC, USA). The statistical model included fixed effects of treatment, time (sampling date), treatment×time interaction, and the random effect of the animal. Before analyses, all data were screened for normality using the UNIVARIATE procedure in SAS. Data that were not normally distributed were log-transformed. Three variance–covariance structures (auto-regressive type 1, compound symmetry, and Toeplitz) were tested, and the covariance structure that minimized the Schwarz information criterion was chosen. Initial BW was used as a covariate for final weight. Growth performance data were analyzed using one-way analysis of variance. The Tukey–Kramer test was used for comparisons among treatments at the same time point or for examining changes over time within the same group. The threshold of significance was set at p≤0.05; trends were declared at 0.05<p≤0.10.

## RESULTS AND DISCUSSION

### Growth performance

Neither transportation nor the administration of meloxicam and/or Cr in transported groups affected (p = 0.99) average daily gain and feed intake (Table 3). A previous study found that meloxicam administration prevented growth performance losses caused by long-distance transportation (1,440 km) via trailer in Angus×Hereford steers [19]. Additionally, Cr supplementation improved growth performance of Charolais-crossed steer calves stressed by extended truck transportation (44 h) [10]. Our results suggest that Cr or meloxicam administration in short-distance transportation conditions may not have a beneficial effect on growth performance. No comparable literature for the effects of meloxicam and/or Cr on growth performance after short-distance transportation in cattle was found.

### Blood cortisol and glucose concentrations

**Figure 1:**
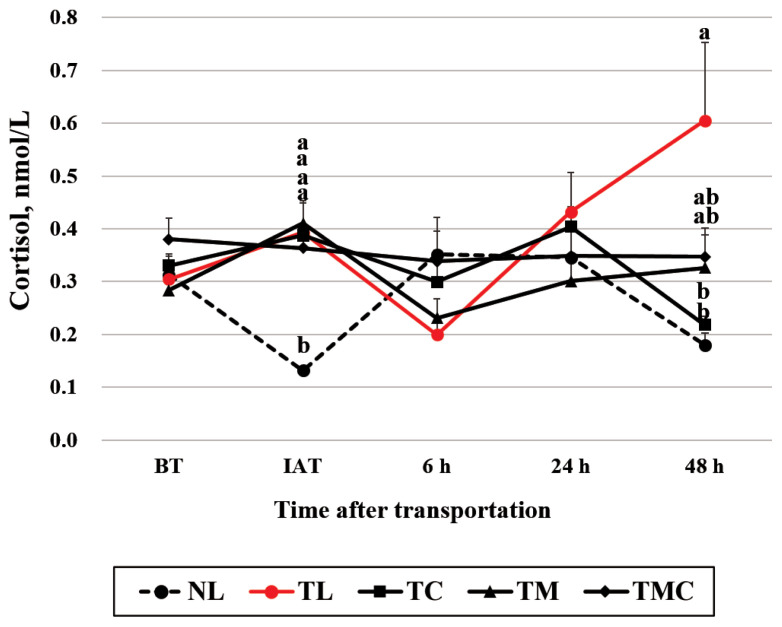
Blood concentrations of cortisol (mean + standard error of the mean, nmol/L) in Holstein heifers transported for 120 km and administered chromium (Cr) and meloxicam. NL, no transportation + lactose monohydrate administration; TL, transportation + lactose monohydrate administration; TC, transportation + Cr administration; TM, transportation + meloxicam administration; TMC, transportation + Cr and meloxicam administration. Blood samples were collected 2.5 h before transportation (BT), immediately after transportation (IAT), and at 6 h, 1 d, and 2 d after transportation. ^a,b^ At each time point, means with different superscripts differ at p<0.05. Treatment effect, p<0.01; time effect, p<0.01; treatment ×time effect, p<0.001.

A treatment×time interaction was detected (p<0.001) for plasma cortisol concentrations (Figure 1). Plasma cortisol concentrations in the NL group (average 0.13 and 0.18 nmol/L) were lower (p<0.05) than in the TL group (average 0.39 and 0.61 nmol/L) at IAT and 48 h after transportation, respectively (Figure 1). These differences were not detected at 6 or 24 h after transportation. Cortisol concentrations in the TL group animals were not changed at IAT but elevated at 48 h after transportation. Consistent with our study, blood cortisol concentrations in a young calf study were not changed IAT but were elevated 1 and 2 d thereafter [20]. In mature cattle, cortisol concentrations were however elevated at IAT and then dropped to baseline within 24 h [21, 22]. Hartmann et al [23] reported that it took a longer time to raise plasma cortisol concentrations in young calves than in older calves. We thus assumed that reason for the elevated cortisol concentrations at 48 h after transportation but not at earlier times in the TL group was because we used relatively young calves (average age 5.5 months) in this study.

At 48 h after transportation, cortisol concentrations were lower (p<0.05) in the TC group (average 0.22 nmol/L) than in the TL group (average 0.61 nmol/L), and TC calves had similar cortisol concentrations to NL calves. These results indicate that Cr administration was effective at alleviating cortisol levels in transported animals, although it was not effective at other times (IAT, 6 h, and 24 h). Similarly, Cr supplementation has been reported to decrease serum cortisol concentrations in Charolais-crossed feeder calves after transportation [10,24]. Cr administration also reduced blood cortisol concentrations in lactating cattle under heat stress conditions [12]. In this study, cortisol concentrations were reduced in the Cr treatment, but not in the combined treatment of Cr and meloxicam, and this inconsistent result requires further study. Dietary Cr may enhance insulin activity [25]. It is unclear whether Cr administration affected insulin activity in this study since serum glucose concentrations were not affected by Cr treatment at 48 h after transportation, although TMC group has low glucose concentrations compared to TL group (Table 4). In this study, repeated blood sampling may cause the stress, and we have thought that the effect of the blood collection method would have been offset, since blood of all animal was collected by the same method. However, there may be a possibility that cortisol concentrations are influenced by the blood sampling effect, in addition to Cr and meloxicam administration effects. Meanwhile, half-life of oral meloxicam was reported to be ranged between 20 and 43 hours [16], suggesting that effect of meloxicam administration would last 2 days.

The TM (average: 0.41 and 0.33 nmol/L, respectively) and TMC (average 0.36 and 0.35 nmol/L, respectively) groups had similar cortisol concentrations (p>0.05) to the TL group, indicating that meloxicam or combined administration of Cr and meloxicam were not effective at alleviating cortisol levels IAT or 48 h after transportation. This was an unexpected result. Nevertheless, our data are consistent with previous results showing that meloxicam administration did not affect plasma cortisol concentration after 1,440 km of transportation in Angus×Hereford steers [19]. Van Engen et al [15] also observed no significant difference in serum cortisol between a control group and a meloxicam administration group after 1,316 km of transportation in Brahman and Angus× Brahman crossbred steers. Interpretation of this study with short-distance transportation has a limitation, since most of the transport studies having meloxicam and/or Cr treatment have been dealt with long-duration, as described above.

### Animal behavior

**Figure 2:**
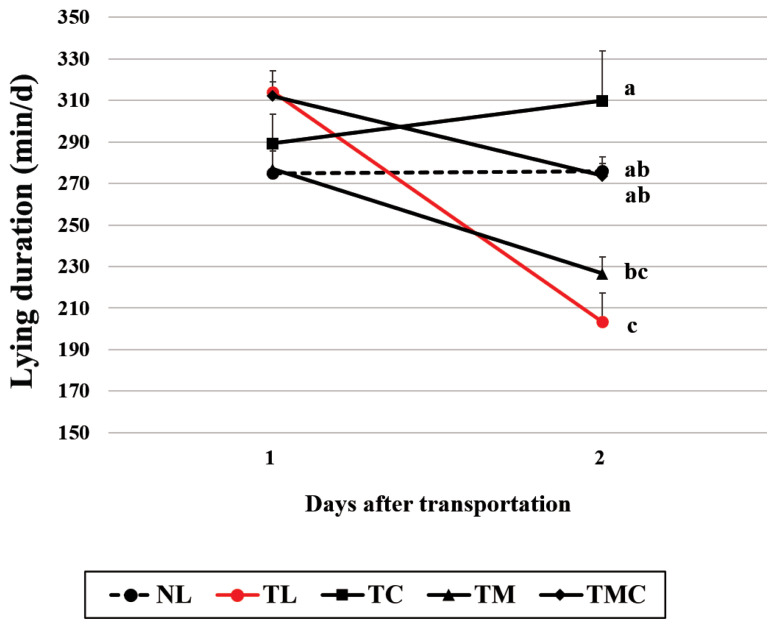
Lying duration (min/d) in Holstein heifers transported 120 km and administered chromium (Cr) and meloxicam. NL, no transportation + lactose monohydrate administration; TL, transportation +lactose monohydrate administration; TC, transportation + Cr administration; TM, transportation + meloxicam administration; TMC, transportation + Cr and meloxicam administration. Lying behavior were monitored for the first 2 days after transportation. a-c At each time point, means with different superscripts differ at p<0.05. Treatment effect, p<0.01; time effect, p<0.01; treatment×time effect, p<0.001.

Lying duration (min/d) was shorter (p<0.05) in the TL group than in the NL group at 2 d after transportation, but not at 1 d (treatment×time effect, p<0.01; Figure 2), indicating that transportation decreased lying duration. Our cortisol data indicate that transported-non-treated heifers has suffered from the stress at 2 d after transportation. Thus, the decreased lying duration in transported-non-treated heifers at 2 d could be in part explained by the higher circulating cortisol concentrations compared to non-transported heifers.

Lying duration was longer (p<0.05) in the TC and TMC groups than in TL group at 2 d after transportation, but not at 1 d. Our results demonstrate that Cr administration in transported calves improves lying behavior at 2 d after transportation. As described above, we found that transportation elevated cortisol concentrations and that Cr administration reduced the elevated cortisol concentrations in transported calves. Thus, decreased transportation stress by Cr administration may help improve lying behavior. However, meloxicam administration did not affect (p>0.05) lying duration in transported calves at 1 d or 2 d after transportation, possibly because it did not change cortisol concentrations.

## CONCLUSION

Road transportation increased plasma cortisol concentrations and decreased lying duration without affecting average daily gain or feed intake over 2 weeks. Cr administration reduced cortisol concentrations and improved lying behavior. Cr administration before transportation may be a viable strategy to alleviate road transportation-induced stress.

## Figures and Tables

**Table 1:** Ingredients and chemical compositions of total mixed ration

Item	Percentage (dry matter)
Ingredient	
Corn	1.00
Corn gluten feed	9.00
Alfalfa hay	4.00
Oats	1.04
Ryegrass silage	32.00
Probiotics	0.15
Vitamin premix^1)^	0.15
Tall fescue, hay	6.00
Beet pulp	5.00
Brewers grain	7.00
Rice straw	8.11
Concentrate^2)^	26.55
Chemical composition	
Dry matter	64.08
Crude protein	10.20
Ether extract	2.06
Crude fiber	11.42
Crude ash	5.58
Neutral detergent fiber	30.50
Acid detergent fiber	17.49
Non-structure carbohydrate	17.71
Total digestible nutrient	77.92
Digestible energy^3)^ (Mcal/kg)	1.96
Metabolizable energy^4)^ (Mcal/kg)	1.53

1) Vitamin premix contained 5,000,000 IU vitamin A; 1,500,000 IU vitamin D_3_; 30,000 IU vitamin E; 2250 mg Cu; 37,500 mg Fe; 21,000 mg Mn; 75 mg Co; 35,000 mg Zn; 450 mg KIO_3_; 70 mg Na_2_SeO_3_; 15,000 mg Zn-methionine; 30 mg Se yeast; 9,000 mg chelated Zn; 525 mg chelated Cu; 10,000 mg niacin; 10,000 mg pantothenic acid; and 100 mg biotin per kg of additive (provided by Nonghyupsaryo, Busan, Korea).

2) Detailed composition of the concentrate is provided in Table 2.

3) Digestible energy = 0.04409×total digestible nutrient (%).

4) Metabolizable energy = [1.01×(digestible energy)−0.45]+0.0046× (ether extract−3) [26].

**Table 2:** Ingredients and chemical composition of concentrate

Item	Percentage (dry matter)
Ingredient	
Corn	19.16
Dried distiller’s grains with solubles	7.00
Palm kernel cake	10.00
Corn gluten feed	17.00
Tapioca	5.00
Soybean meal	13.00
Molasses	5.00
Calcium phosphate	0.23
Protease	0.10
Calcium sulfate	0.20
Wheat bran	0.90
Coconut meal	16.00
Corn germ meal	4.00
Limestone	2.00
Salts	0.40
Chemical composition	
Dry matter	89.47
Crude protein	18.50
Ether extract	3.46
Crude fiber	8.30
Crude ash	7.40
Neutral detergent fiber	26.20
Acid detergent fiber	12.47
Non-structure carbohydrate	36.59
Total digestible nutrient	72.00
Digestible energy^1)^ (Macl/kg)	3.17
Metabolizable energy^2)^ (Mcal/kg)	2.77

1) Digestible energy = 0.04409×total digestible nutrient (%).

2) Metabolizable energy = [1.01×(digestible energy)−0.45]+0.0046× (ether extract−3) [26].

**Table 3:** Effects of transportation and chromium and meloxicam administration on the growth performance of Holstein calves over 2 weeks

Item	NL^1)^	TL^1)^	TC^1)^	TM^1)^	TMC^1)^	SEM	p-value
Initial body weight^2)^ (kg)	170	172	172	175	172	29.6	0.99
Final body weight^3)^ (kg)	188	191	190	191	188	30.2	0.99
Average daily gain (kg)	1.30	1.40	1.27	1.21	1.16	0.26	0.28
Feed intake^4)^ (kg/calf/d)	7.89	7.89	7.84	7.83	7.88	0.02	-
Gain:feed	0.165	0.177	0.162	0.154	0.147	0.03	-

N = 10 per group except feed intake and gain/feed ratio, where n = 2 per group.

SEM, standard error of the mean.

1) NL, no transportation + lactose monohydrate administration; TL, transportation + lactose monohydrate administration; TC, transportation + Cr administration; TM, transportation + meloxicam administration; TMC, transportation + Cr and meloxicam administration.

2) Initial body weight at −1 d.

3) Final body weight at 14 d.

4) Intake was measured pen-base (5 calves/pen, 2 pens/group); intake/calf was calculated by dividing the pen intake by 5 calves.

**Table 4:** Effect of transportation and administration of meloxicam and chromium on serum glucose concentrations (mg/dL) in young Holstein heifers

Item	Treatment^1)^					SEM	p-value		
	
	NL	TL	TC	TM	TMC		Treatment	Time	Treatment×time
Glucose (mg/dL)							0.77	<0.001	<0.001
BT	67.6	69.2	73.7	75.3	74.1	2.32			
IAT	66.2	63.2	63.3	59.8	66.4	2.41			
6 h	72.7	75.4	73.5	78.9	74.5	2.22			
24 h	64.8	65.7	63.1	60.0	56.3	2.96			
48 h	76.4^a^	76.5^a^	75.4^ab^	68.8^ab^	64.2^b^	2.41			

SEM, standard error of the mean; BT, before transportation; IAT, immediately after transportation.

1) Completely randomized design using the MIXED procedure: NL, no transportation + lactose monohydrate administration; TL, transportation + lactose monohydrate administration; TC, transportation + Cr administration; TM, transportation + meloxicam administration; TMC, transportation + Cr and meloxicam administration.

a,b At each time point, means with different superscripts differ at p<0.05.

Blood samples were collected 2.5 h BT, IAT, and at 6 h, 1 d, and 2 d after transportation.
